# Long-Term Results of Immunogenicity of Booster Vaccination against SARS-CoV-2 (Hybrid COV-RAPEL TR Study) in Turkiye: A Double-Blind, Randomized, Controlled, Multicenter Phase 2 Clinical Study

**DOI:** 10.3390/vaccines11071234

**Published:** 2023-07-12

**Authors:** Ihsan Ates, Ayse Batirel, Mehtap Aydin, Fatma Yilmaz Karadag, Abdulsamet Erden, Orhan Kucuksahin, Berkan Armagan, Serdar Can Guven, Ozlem Karakas, Selim Gokdemir, Lutfiye Nilsun Altunal, Aslihan Ayse Buber, Emin Gemcioglu, Oguzhan Zengin, Osman Inan, Enes Seyda Sahiner, Gulay Korukluoglu, Zafer Sezer, Aykut Ozdarendeli, Ahmet Omma, Ates Kara

**Affiliations:** 1 Department of Internal Medicine, University of Health Sciences, Ankara City Hospital, 06800 Ankara, Türkiye; 2 Department of Infectious Diseases and Clinical Microbiology, University of Health Sciences, International Medical School, Kartal Dr. Lutfi Kirdar City Hospital, 34865 Istanbul, Türkiye; 3 Department of Infectious Diseases and Clinical Microbiology, University of Health Sciences, Umraniye Training and Research Hospital, 34760 Istanbul, Türkiye; 4 Department of Infectious Diseases, University of Health Sciences, Sancaktepe Sehit Prof. Dr. Ilhan Varank Training and Research Hospital, 34785 Istanbul, Türkiye; 5 Clinic of Rheumatology, Ankara City Hospital, 06800 Ankara, Türkiyeberkanarmagan@gmail.com (B.A.);; 6 Department of Clinical Pharmacology, University of Health Sciences, Kartal Dr. Lutfi Kirdar City Hospital, 34865 Istanbul, Türkiye; 7 Virology Laboratory, General Directorate of Public Health, 06560 Ankara, Türkiye; 8 Department of Pharmacology, Erciyes University, 38030 Kayseri, Türkiye; 9 Vaccine Research, Development and Application Center, Erciyes University, 38280 Kayseri, Türkiye; 10 Department of Microbiology, Medical Faculty, Erciyes University, 38030 Kayseri, Türkiye; 11 Clinic of Rheumatology, University of Health Sciences, Ankara City Hospital, 06800 Ankara, Türkiye; 12 Division of Pediatric Infectious Diseases, Department of Pediatrics, Hacettepe University, 06230 Ankara, Türkiye; 13 Türkiye Vaccine Institute, 06270 Ankara, Türkiye

**Keywords:** TURKOVAC, CoronaVac, booster dose, immunogenicity, long-term results

## Abstract

The immunogenicity of vaccines decreases over time, causing a need for booster doses. This study aimed to present the long-term (Day 84) immunogenicity results of the double-blind, randomized, controlled, phase II Hybrid COV-RAPEL TR Study (NCT04979949), in which the TURKOVAC or CoronaVac vaccines were used as a booster after the second dose of primary vaccination with CoronaVac. A total of 190 participants from the Hybrid COV-RAPEL TR Study, who had both Day 28 and Day 84 immunogenicity results, were included. The immunogenicity on Day 84, regarding the neutralizing antibody positivity (Wuhan and Delta variants) and anti-spike immunoglobulin (Ig) G (IgG) antibody positivity, was compared between TURKOVAC and CoronaVac vaccine arms according to sex and age groups. Overall, antibody positivity showed a slight decrease on Day 84 vs. Day 28, but was not different between TURKOVAC and CoronaVac arms either for sexes or for age groups. However, TURKOVAC produced better antibody response against the Delta variant than CoronaVac, while CoronaVac was superior over TURKOVAC regarding neutralizing antibody positivity in the 50–60 years age group, regardless of the variant. A single booster dose, after the completion of the primary vaccination, increases antibody positivity on Day 28 which persists until Day 84 with a slight decrease. However, an additional booster dose may be required thereafter, since the decrease in antibody titer may be faster over time.

## 1. Introduction

The coronavirus disease of 2019 (COVID-19) was first reported, in December 2019 in Wuhan, China, as pneumonia cases of unknown etiology with human-to-human transmission that were not responding to standard therapies. The disease then spread rapidly across the world and the outbreak was declared as a Public Health Emergency of International Concern (PHEIC) on 22 January 2020, and then was announced by the World Health organization (WHO) as a pandemic on 11 March 2020 [1]. Since that time, the COVID-19 pandemic has been affecting the whole world. The initial variant of concern (VOC) was the SARS-CoV-2 alpha variant, which was then followed by the Delta (June 2021) and Omicron (November 2021) variants [1]. From the beginning of the pandemic, many countries started trials to develop a vaccine against the virus, and vaccine trials continued and accelerated with these emerging new variants. Numerous vaccines of various types have been developed so far and, as of 2 December 2022, there were 242 vaccine candidates, 821 vaccine trials, and 50 vaccines that have been approved in at least one country [2]. There were also 11 vaccines that were granted Emergency Use Listing (EUL) by the WHO, including the following 4 types: protein subunit vaccines (such as Nuvaxovid [Novavax], Serum Institute of India); COVOVAX (Novavax formulation; Serum Institute of India); RNA vaccines (such as Comirnaty [Pfizer/BioNTech]; Spikevax [Moderna]); non-replicating viral vector vaccines (such as Jcovden [Janssen (Johnson & Johnson)]; Vaxzevria [Oxford/AstraZeneca]); inactivated vaccines (such as Covaxin [Bharat Biotech]; and CoronaVac [Sinovac]) [2]. Despite the significant number of deaths, the rapid development of COVID-19 vaccines reduced human-to-human transmission of infection and decelerated disease spread, resulting in millions of lives saved worldwide [3]. As of 28 May 2023, a total of 13,356,281,548 doses of vaccine have been administered globally [4].

During the pandemic, primary vaccination in Türkiye was started first on 13 January 2021 with the inactivated CoronaVac vaccine in people aged ≥ 65 years, in healthcare professionals and in high-risk people. On 2 April 2021, the mRNA vaccine (BNT162B2) was included in the primary vaccination schedule and a booster dose was recommended to be administered six months after the second dose of primary vaccination. As of 28 January 2023, a total of 139,694,693 vaccine doses have been administered in Türkiye [5].

As in many other countries in the world, vaccine development studies for COVID-19 in Türkiye started with the pandemic [6]. In December 2021, the inactivated SARS-CoV-2 vaccine—TURKOVAC, which has been produced by SBT Science and Biotechnologies and manufactured by Kocak Farma (Koçak Farma Production Facilities, Tekirdag, Türkiye), was authorized for emergency use by the Turkish Medicines and Medical Devices Agency [7]. TURKOVAC is an inactivated whole virion vaccine containing the hCoV-19 strain of SARS-CoV-2. The investigation of the preclinical immunogenicity, efficacy, and safety of TURKOVAC in an animal model of COVID-19 showed no safety issues but improved humoral immune responses and lowered the incidence of upper respiratory tract infection induced by SARS-CoV-2 [8]. 

The advantages of inactivated vaccines include easy storage and transport, which make them more available particularly for developing countries [9]. In addition to these advantages, studies from various countries demonstrated favorable efficacy with good safety profiles for immunization against COVID-19 [6,10,11,12].

In general, inactivated vaccines showed protective effects 14 days after the second dose [13]. It has been reported that protective neutralizing antibody titers decrease at 6 months in patients who have had COVID-19 infection, suggesting that a complete primary vaccination would also show similar efficacy [14]. Nevertheless, booster vaccination results in higher antibody titers and provides a longer period of protection [15,16]. Despite the lack of definite data about the timing of the booster dose, it is recommended 4–6 months after the second dose of primary vaccination [17].

The present study aimed to present the long-term immunogenicity results of the double-blind, randomized, controlled, phase II Hybrid COV-RAPEL TR Study [10], in which the TURKOVAC or CoronaVac vaccine was used for boosting after the second dose of primary vaccination with CoronaVac.

## 2. Materials and Methods

Volunteers with both Day 28 and Day 84 immunogenicity results from the double-blind, randomized, controlled, phase II study (Hybrid COV-RAPEL TR Study; ClinicalTrials.gov; Identifier: NCT04979949) were analyzed to evaluate the long-term (Day 84) immunogenicity results of a single booster dose of CoronaVac and TURKOVAC vaccines. The Method section of the Hybrid COV-RAPEL TR study was detailed in the article published by Omma et al. [10].

Briefly, this study started on 12 July 2021 in Ankara, Türkiye as a single-center study and was continued as a multicenter study. The study included healthy adults of both sexes, aged 18–60 years, who were vaccinated with two doses of CoronaVac vaccine and wanted to receive a booster dose of COVID-19 vaccine, and did not exceed the duration of at least 90 days and a maximum of 270 days from the second dose of vaccination. Mild/moderate well-controlled comorbidities were allowed. The main exclusion criteria were: acute illness or fever within 48 h or antipyretic/analgesic medication use within 24 h before planned administration of vaccine; pregnancy at the time of enrollment or planning to become pregnant within the first 3 months following vaccination; breast-feeding; having a known history of SARS-CoV-2; having had COVID-19 after primary vaccination; receiving any vaccine (licensed or investigational) other than the study intervention pre and post 30 days of each study vaccine (one week for licensed seasonal flu vaccine or pneumococcal vaccine); and severe disease, disorder, or a finding (severe and/or uncontrolled cardiovascular disease, respiratory disease, gastrointestinal disease, liver disease, kidney disease, endocrine disorder, and neurological disease) that could significantly increase the subject’s risk for participation in the study, affect the subject’s ability to participate in the study, or impair the interpretation of study data. (Please refer to the study by Omma et al. [10] for further details of inclusion and exclusion criteria).

Among 236 eligible patients, 222 volunteers were randomly assigned in the Hybrid COV-RAPEL TR Study between 12 July 2021 and 10 September 2021 in a 1:1 ratio to receive either a single booster dose of TURKOVAC or CoronaVac vaccine. Age and sex quotas were used for patient selection (Table 1). The randomization was conducted using the Omega Research Randomization and Investigational Product Management System (Omega Interactive Voice Response Systems [IVRS]/Interactive Web Response Systems [IWRS]). The study (clinical trial protocol and informed consent forms) was approved by the Ethics Committee of Ankara City Hospital (No: E2-21-640, Date: 22 June 2021). The full trial protocol can be accessed through the publication by Omma et al. [10].

### 2.1. Procedures

Either CoronaVac or TURKOVAC vaccine was used for the booster dose. Both CoronaVac and TURKOVAC are available in pre-filled syringes or vials; for both vaccines, dosage is 3 μg/0.5 mL per injection. 

CoronaVac vaccine (Vero Cell), which is manufactured by Sinovac Life Sciences Company, Beijing, China using CZ02 Strain, contains 600 SU (3 μg) of SARS-CoV-2 antigen as the inactivated active ingredient, and 0.225 mg of aluminum hydroxide as adjuvant in a 0.5 mL milky-white aqueous suspension without preservatives or stabilizers [18].

TURKOVAC vaccine, which was manufactured by Kocak Farma (Koçak Farma Production Facilities, Tekirdag, Türkiye) using hCoV-19 strain (hCoV-19/Turkiye/ERAGEM-001/2020 strain, GenBank accession number; MT327745.1 and GISAID; EPI_ISL_424366), contains 600 SU (3 μg) of SARS-CoV-2 antigen as the inactivated active ingredient and Alum Gel (10% AlOH–InvivoGen, San Diego, CA, USA) of 0.50 mg as adjuvant in a 0.5 mL milky-white suspension without any preservatives or stabilizers. Detailed information about manufacturing of TURKOVAC has been presented previously [7,19].

All participants underwent Real Time Reverse Transcriptase Polymerase Chain Reaction (RT-PCR) testing before the booster dose. Volunteers with positive pre-vaccine PCR test were excluded. Physical examination was performed and vital signs were recorded. A single dose of booster vaccine (TURKOVAC or CoronaVac) was injected into the upper arm deltoid muscles (preferably left) of the volunteers. All except the study pharmacist preparing the product, the study team and volunteers, including the nurse who performed the vaccination, were blinded.

Blood was drawn for immunogenicity analysis at the study visit on day 0 (the day of vaccination or 1 day before the vaccination). The amount of changes in SARS-CoV-2 neutralizing antibody and anti-spike immunoglobulin G (IgG) was evaluated in both groups on Day 28 (±2 days) and on Day 84 after the booster dose.

A virus neutralization test technique was used for SARS-CoV-2 neutralizing antibody detection. The neutralizing antibody titers against Wuhan and Delta variants were evaluated according to the threshold values of 1/6 and 1/12 [20].

### 2.2. Reverse Transcriptase Polymerase Chain Reaction

Detection of SARS-CoV-2 in nasopharyngeal/oropharyngeal samples was performed by RT-PCR method for SARS-CoV-2 specific ‘Orf1ab’ and ‘N’ genes targeting human ‘RNaseP (Ribonuclease P)’ genes. RNaseP was used as internal control to evaluate sample-based inhibition control and kit reagent control.

Sterile nylon, dacron or rayon swabs with flexible plastic shafts were used to collect nasopharyngeal/oropharyngeal samples from suspected COVID-19-positive patients. Swabs were placed in vNAT (Viral Nucleic Acid buffer; various manufacturers) transfer tubes, vNAT transfer tubes containing Tween-20 (to protect RNA), NaN3 (to protect RNA), BSA (to improve PCR reaction), and Polyethyleneimine-coated tetradecyldimethyl benzyl ammonium chloride-based nanoparticles (for isolation and RNA protection). Nucleic acid isolation process occurs during transfer with these tubes. The samples accepted to the laboratory were put into the PCR process after vortexing for 15 s. Samples were transported and tested within two hours after collection.

RT-PCR was performed using Coronex-COVID19 (Ver. 2.0) Multipleks RT-qPCR Diagnosis Kit (DS Bio and Nano Technology, Ankara, Türkiye).

The 20 μL reaction mix contained 5 μL of RNA, 12.5 μL of CORONEX-COVID 19 DS Mix E (RT-qPCR master mix) and 2.5 μL of CORONEX-COVID 19 DS PP1 primer and prob mix (Orf1ab and N genes for SARS-CoV-2 detection, Rnase P gene for internal control). Positive control for amplification control and no-template control, to assess contamination, were also used in each run. Thermal cycling was performed at 48 °C for 20 min for reverse transcription, followed by 95 °C for 5 min and then 35 cycles of 95 °C for 5 s, and 60 °C for 10 s, in Rotor-Gene Q device (Qiagen, Hilden, Germany). Cycle threshold (Ct) values of less than 33 were defined as positive.

### 2.3. Viral Isolation and Microneutralization Test Technique

Virus isolation procedure and microneutralization test technique are described in detail in the article published by Omma et al. [10]. Briefly, Wuhan and Delta strains were used to detect the neutralizing antibody of vaccinated sera in order to evaluate the neutralizing ability of vaccine immunization. The virus name, accession ID, and date of the collection for the Wuhan and Delta strain were as follows: hCoV-19/Türkiye/HSGM-1192/2020, EPI_ISL_811143, and 2020, respectively, for the Wuhan variant and hCoV-19/Türkiye/HSGM-B18515/2021, EPI_ISL_2958539, and 2021, respectively, for the Delta variant.

Serum samples were studied by microneutralization test (MNT) at the General Directorate of Public Health, National Virology Reference Laboratory. This step has been performed with the Wuhan and Delta strains concurrently in different cell culture microplates. The neutralization endpoint titer was determined as the highest serum dilution inhibiting the virus infection in 50% of the inoculated wells [21]. The MNT titer ≥ 4 was considered positive. The test was checked for virus and cell control with a phase contrast cell culture microscope and was evaluated as positive when 100% of SARS-CoV-2-specific cytopathic effect (CPE) was observed in the virus control section.

### 2.4. Measurement of Anti-Spike IgG Level

This procedure is also described in detail in the article by Omma et al. [10]. Briefly, a fully automated two-step sandwich immunoassay, using indirect chemiluminescent technology, was performed to measure anti-spike IgG levels (Atellica IM sCOVG Assay, Siemens Healthineers, Erlangen, Germany). There is a direct relationship between the amount of circulating SARS-CoV-2 IgG antibody and the amount of relative light units detected by the system. The analytical assay range of 0.50–150.00 index is reported as nonreactive (<1.00 index) or reactive (≥1.00 index).

While SARS-CoV-2 PCR tests were performed at each center, anti-spike protein IgG test was performed at the Ankara City Hospital Medical Microbiology Clinic Laboratory. SARS-CoV-2 neutralizing antibody tests were conducted in the National Virology Reference Laboratory of the Turkish Ministry of Health, General Directorate of Public Health. 

### 2.5. Statistical Analysis

Data analyses were performed using the PASW Statistics for Windows, Version 18.0. (SPSS Inc., Chicago, IL, USA). Statistical significance was set at a *p* value of <0.05. The detailed expression of statistical analyses (sample size calculation) of the Hybrid COV-RAPEL TR Study was presented in the study by Omma et al. [10]. In brief, descriptive statistics were expressed as numbers and percentages for categorical variables and mean, standard deviation, and median, and interquartile range (IQR) for numerical variables. Visual (histogram and probability graphs) and analytical methods (Kolmogorov–Smirnov/Shapiro–Wilk tests) were used to test normality of data. The amount of SARS-CoV-2 neutralizing antibody and SARS-CoV-2 anti-spike IgG antibody on days 0, 28, and 84 were presented descriptively.

## 3. Results

Among the 222 volunteers evaluated in the Hybrid COV-RAPEL TR Study, 190 volunteers (88 in the TURKOVAC arm and 102 in the CoronaVac arm) had both Day 28 and Day 84 results. The first 28th day safety and immunogenicity results of a single booster dose of CoronaVac or TURKOVAC vaccines were reported by Omma et al. [10].

The two vaccine arms were comparable in terms of the distribution of the participants among sexes (*p* = 0.768), age groups (*p* = 0.587), and COVID-19 (+) subjects (*p* = 0.391) (Table 2). Until Day 84 after the booster dose, the number of COVID-19 (+) participants were same in each vaccine arm; however, it was higher in the TURKOVAC arm after Day 84 (Table 2).

**Figure 1:**
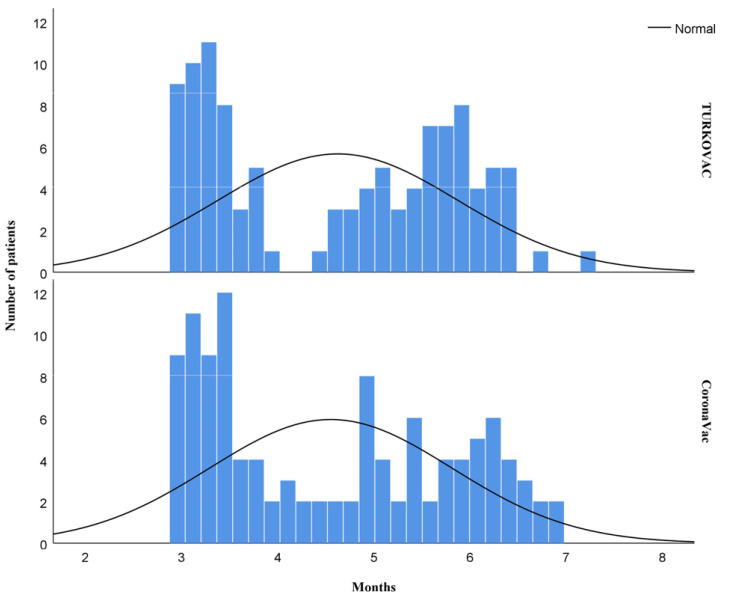
The time between the second dose and the booster dose in the TURKOVAC and CoronaVac vaccine arms.

The time span between the second dose of the primary vaccination and the booster dose was similar between the two groups (Figure 1).

**Figure 2:**
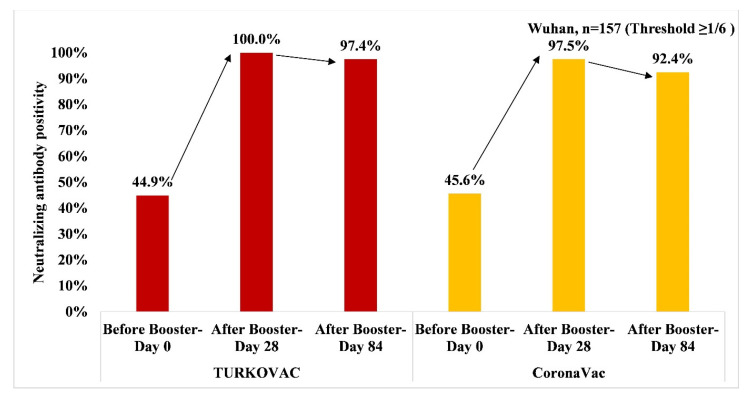
Neutralizing antibody positivity against the Wuhan variant in TURKOVAC and CoronaVac vaccine arms at the threshold value of ≥1/6.

The neutralizing antibody positivity against the Wuhan variant slightly decreased on Day 84 vs. Day 28 in both vaccine arms at the threshold value of 1/6 (Figure 2). The two vaccine arms were comparable in terms of antibody positivity on Day 84. The neutralizing antibody positivity against the Wuhan variant at the threshold value of 1/12 is given in the Supplementary Material S1 (Figure S1).

**Figure 3:**
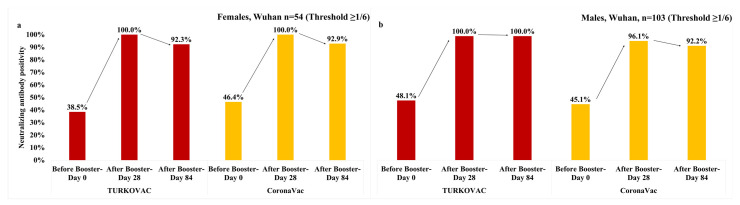
Neutralizing antibody positivity against the Wuhan variant in the TURKOVAC and CoronaVac vaccine arms at the threshold value of ≥1/6: (**a**) in females; (**b**) in males.

In females, the neutralizing antibody positivity against the Wuhan variant was slightly decreased on Day 84 vs. Day 28 in both vaccine arms at the threshold value of 1/6 (Figure 3a). The neutralizing antibody positivity against the Wuhan variant in males reached the highest level on Day 28 in both vaccine arms; while the antibody positivity persisted on Day 84 in the TURKOVAC arm, it decreased slightly in the CoronaVac arm at the threshold value of 1/6 (Figure 3b). The neutralizing antibody positivity against the Wuhan variant at the threshold value of ≥1/12 is given in the Supplementary Material S1 (Figure S2).

**Figure 4:**
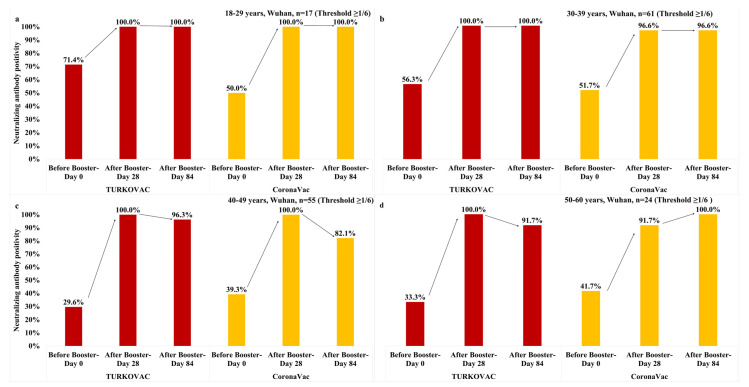
Neutralizing antibody positivity against the Wuhan variant in the TURKOVAC and CoronaVac vaccine arms at the threshold value of 1/6 in the (**a**) 18–29 years age group; (**b**) 30–39 years age group; (**c**) 40–49 years age group; and (**d**) 50–60 years age group.

Regarding the age groups, the neutralizing antibody positivity against the Wuhan variant at the threshold value of 1/6 was maximum (100%) on Day 28 and persisted on Day 84 in both vaccine arms in the 18–29 years age group (Figure 4a). In the 30–39 years age group, maximum (100%) and nearly maximum (96.6%) positivity rates were achieved on Day 28 in the TURKOVAC arm and CoronaVac arm, respectively, and persisted on Day 84 in both vaccine arms (Figure 4b). In the 40–49 years age group, maximum positivity rates were achieved on Day 28, which then slightly decreased on Day 84 in both vaccine arms (Figure 4c). In the 50–60 years age group, the maximum positivity rate was achieved on Day 28 but then slightly decreased on Day 84 in the TURKOVAC arm, whereas the positivity rate increased on Day 28 and then reached the maximum level (100%) on Day 84 in the CoronaVac arm (Figure 4d). The neutralizing antibody positivity against the Wuhan variant at the threshold value of 1/12 according to the age groups is given in the Supplementary Material S1 (Figure S3).

**Figure 5:**
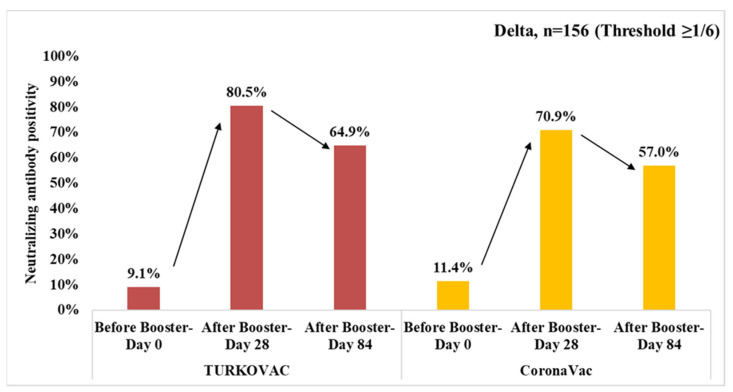
Neutralizing antibody positivity against the Delta variant in the TURKOVAC and CoronaVac vaccine arms at the threshold value of ≥1/6.

The neutralizing antibody positivity against the Delta variant decreased on Day 84 vs. Day 28 in both vaccine arms at the threshold value of 1/6 (Figure 5). The neutralizing antibody positivity against the Delta variant at the threshold value of ≥1/12 is given in the Supplementary Material S2 (Figure S4).

**Figure 6:**
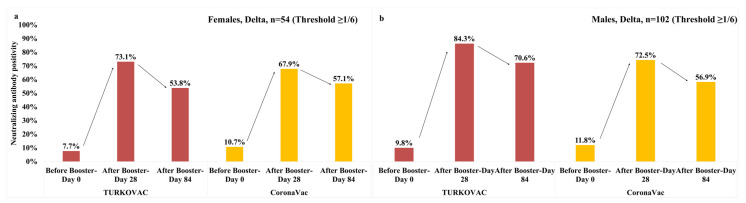
Neutralizing antibody positivity against the Delta variant in the TURKOVAC and CoronaVac vaccine arms at the threshold value of ≥1/6: (**a**) in females; (**b**) in males.

At the threshold value of ≥1/6, the neutralizing antibody positivity against the Delta variant showed a decreasing trend until Day 84 in both vaccine arms and both sexes; however, the positivity on Day 84 was higher in males in the TURKOVAC arm (Figure 6a,b). The neutralizing antibody positivity against the Delta variant at the threshold value of 1/12 is given according to sexes in the Supplementary Material S2 (Figure S5).

**Figure 7:**
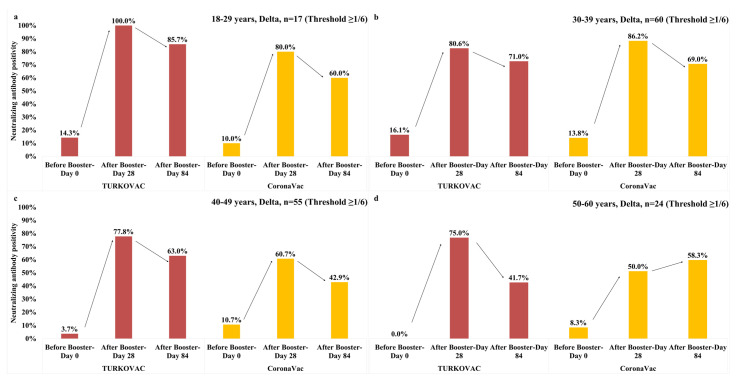
Neutralizing antibody positivity against the Delta variant in the TURKOVAC and CoronaVac vaccine arms at the threshold value of ≥1/6 in the (**a**) 18–29 years age group; (**b**) 30–39 years age group; (**c**) 40–49 years age group; and (**d**) 50–60 years age group.

The neutralizing antibody positivity against the Delta variant at the threshold value of 1/6 according to the vaccine arms and age groups were as follows: in the 18–29, 30–39, and 40–49 years age groups, antibody positivity slightly decreased on Day 84 vs. Day 28 in both vaccine arms but remained higher in the TURKOVAC arm in all three age groups (Figure 7a–c). In the 50–59 years age group, antibody positivity was decreased on Day 84 vs. Day 28 in the TURKOVAC arm and was lower than the CoronaVac arm, in which the antibody positivity showed an increase on Day 84 (Figure 7d). The neutralizing antibody positivity against the Delta variant at the threshold value of ≥1/12 is given according to age groups in the Supplementary Material S2 (Figure S6).

**Figure 8:**
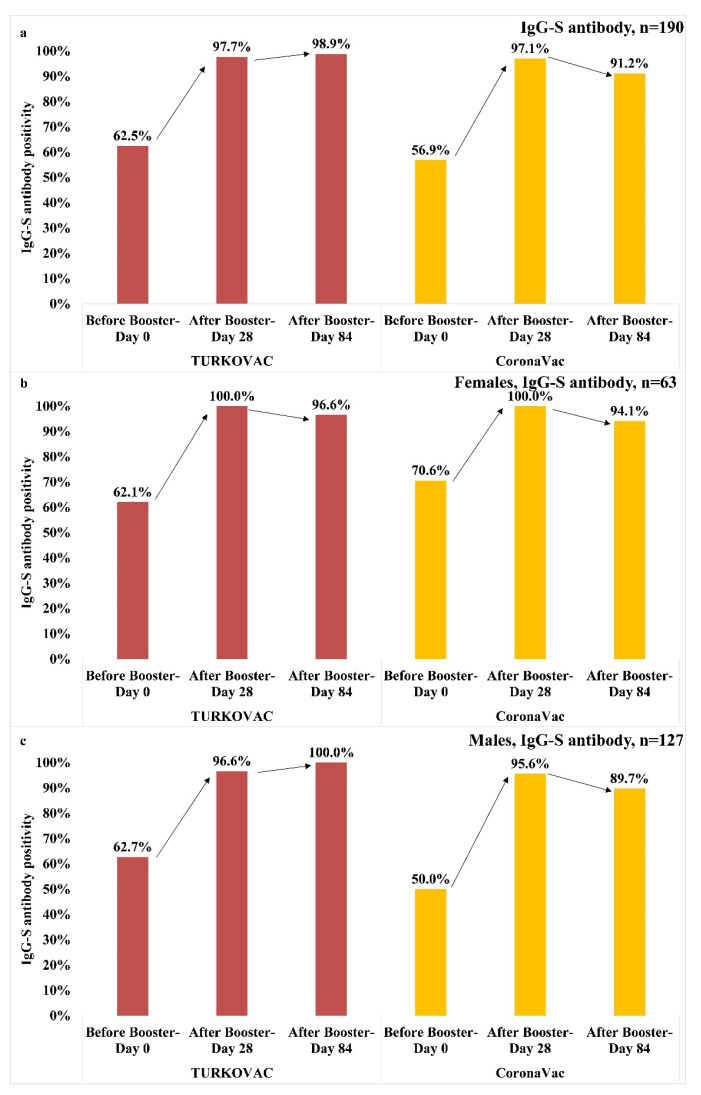
Anti-spike IgG antibody positivity (**a**) in the whole study group; (**b**) in females; and (**c**) in males.

Anti-spike IgG antibody positivity increased from 97.7% on Day 28 to 98.9% on Day 84 in the TURKOVAC arm, but showed a slight decrease from 97.1% on Day 28 to 91.2% on Day 84 in the CoronaVac arm (Figure 8a). Anti-spike IgG antibody positivity in females decreased on Day 84 vs. Day 28 in both vaccine groups; however, anti-spike IgG antibody positivity on Day 84 was higher in the TURKOVAC arm (Figure 8b). In males, anti-spike IgG antibody positivity continued to increase until Day 84 from Day 28 and reached 100% in the TURKOVAC arm, but decreased from 95.6% on Day 28 to 89.7% on Day 84 in the CoronaVac arm (Figure 8c). Anti-spike IgG antibody positivity was higher on Day 84 in the TURKOVAC arm vs. the CoronaVac arm, both in the whole study group and in both sexes.

**Figure 9:**
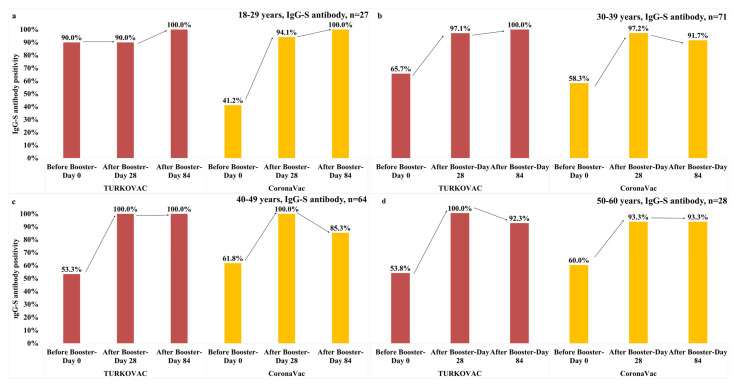
Anti-spike IgG antibody positivity in each vaccine group according to the age groups: (**a**) 18–29 years age group; (**b**) 30–39 years age group; (**c**) 40–49 years age group; and (**d**) 50–60 years age group.

In the 18–29 years age group, anti-spike IgG antibody positivity remained the same on Day 28 as on Day 0 in the TURKOVAC arm, but showed an increase until Day 28 in the CoronaVac arm. However, anti-spike IgG antibody positivity reached the maximum level on Day 84 in both vaccine groups (Figure 9a). In the 30–39 years age group, anti-spike IgG antibody positivity was comparable between vaccine groups on Day 28. However, anti-spike IgG antibody positivity continued increasing and reached the maximum level on Day 84 in the TURKOVAC arm, while it showed a decreasing trend until Day 84 in the CoronaVac arm (Figure 9b). In the 40–49 years age group, anti-spike IgG antibody positivity showed an increasing trend and reached the maximum level on Day 28 in both vaccine arms. While anti-spike IgG antibody positivity remained at the maximum level in the TURKOVAC arm, it showed a decreasing trend until Day 84 in the CoronaVac arm (Figure 9c). In the 50–60 years age group, anti-spike IgG antibody positivity showed an increasing trend after the booster dose and reached the maximum level on Day 28 and then decreased until Day 84 in the TURKOVAC arm, whereas it increased until Day 28 and remained the same on Day 84 in the CoronaVac arm (Figure 9d).

The percentage of participants with a ≥4-fold increase in the anti-spike IgG antibody titers 28 days after the booster dose was higher in the TURKOVAC arm than in the CoronaVac arm (82.5% vs. 64.9%). In both vaccine arms, a higher percentage of females (90.9% in the TURKOVAC arm and 71.1% in the CoronaVac arm; *p* = 0.036) had a ≥4-fold increase in the anti-spike IgG antibody titers as compared to males (78.6% in the TURKOVAC arm and 61.8% in the CoronaVac arm; *p* = 0.028) (Table 3).

## 4. Discussion

It is known that vaccines are the major tools for the prevention of diseases, including COVID-19. To date, numerous vaccine studies across the world demonstrated the efficacy of primary vaccination with two doses of any type of vaccine against COVID-19 [22].

Studies have demonstrated that the immunogenicity of vaccines decreases both over time and due to emerging new variants, with the lowest levels reached at nearly the sixth month after the second dose, suggesting the need for a booster dose [15,23,24]. 

In general, the WHO recommends the primary vaccination for COVID-19 rather than booster doses, both because it is more protective for the whole population and because primary vaccination has not been completed yet, particularly in developing countries [25]. 

Whether booster doses are necessary for the whole population and, if so, to whom and when the booster dose should be applied, are debatable. In May 2022, the WHO recommended boosters for selected populations at 4–6 months intervals after the second dose of primary vaccination with all kinds of COVID-19 vaccine [26]. The timing of and target population for a booster dose show variations among countries. A modeling study from France reported that booster vaccination should be considered for people with high risk of severe disease and/or transmission [27]. In Türkiye, the Ministry of Health declared that those who had two doses of primary vaccination should receive a booster dose three months after the second dose [6]. As in other countries [27], the third dose in Türkiye also began to be applied first to the elderly individuals and healthcare workers, at least three months after the second dose, regardless of the type of vaccine used for primary vaccination. The third dose became available in November 2021 for all people over the age of 18 years [28,29].

Additional booster doses after initial boosters are being recommended in some countries, including Türkiye. Studies from Israel and Canada reported that the fourth dose of COVID-19 vaccine shows short-term efficacy in people over the age of 60 years with minimal efficacy in younger people [30,31]. 

Recent WHO recommendations concerning COVID-19 vaccination include the integration of COVID-19 vaccination into routine immunization schedules, additional booster doses for high-risk healthcare workers 12 months after the previous booster dose but not routinely for medium-risk people, and additional booster doses for children and adolescents according to the disease burden of this age group in the country [32].

The Hybrid COV-RAPEL TR Study [10], which reported the short-term immunogenicity in the same study population as the present study, demonstrated that the neutralizing antibody positivity remarkably increased on Day 28 after the booster dose, regardless of the type of vaccine, variant, sex, and age, and that the threshold value indicates that the antibody response, which decreased in time after the second dose of primary vaccination, was induced by a single booster dose. Similar results indicating a good immune memory have been reported in the studies from both Türkiye and other countries [6,33,34]. 

In the ZOE COVID study, the effectiveness of both homologous and heterologous boosting was found to be higher than that obtained after the second dose of primary vaccination [35]. Another study reported higher vaccine effectiveness with a heterologous booster dose of inactivated COVID-19 vaccine [36]. The favorable effect of the heterologous booster over a homologous one was supported in a study from Türkiye, which reported higher efficacy with CoronaVac when boosted with an mRNA as compared to the third dose of CoronaVac [37]. Yue et al. [38] reported that a single booster dose of inactivated SARS-CoV-2 vaccine also has an impact on the Delta variant.

In the present study, booster doses with TURKOVAC or CoronaVac showed no difference in terms of neutralizing antibody or IgG-spike antibody positivity on Day 84, indicating a good long-term immunogenicity in both vaccine arms. 

The neutralizing antibody positivity against the Wuhan variant on Day 84 was comparable between vaccine arms, regardless of the threshold value. Although both the TURKOVAC and CoronaVac boosters showed efficacy against the Delta variant with comparable neutralizing antibody positivity, the positivity was lower than that against the Wuhan variant. Despite the lack of difference between the vaccine arms, TURKOVAC was associated with slightly higher antibody positivity against both variants regardless of threshold values. Higher responses induced by TURKOVAC against the Delta variant might be due to the fact that the vaccine was developed later than CoronaVac, after the Delta variant had emerged, as well as to the higher amount of aluminum (0.5 mL per dose in the TURKOVAC vs. 0.225 mL per dose in ten CoronaVac) as Alum Gel used as adjuvant in the TURKOVAC, which is the water-soluble form of aluminum hydroxide and has higher binding capacity resulting from its adsorption rate [39]. 

It has been reported that immune responses to COVID-19 vaccines are influenced by the age, sex, and comorbidities of the individual, as well as the type of vaccine and the timing of vaccination [40,41]. However, in this study, no significant difference was found between the sexes in terms of neutralizing antibody titers after the second dose of primary vaccination [42]. Likewise, in the present study, the neutralizing antibody positivity against the Wuhan variant on Day 84 was comparable between the vaccine arms regardless of threshold values and sexes. The neutralizing antibody positivity against the Delta variant was also comparable between the vaccine arms and sexes with lower positivity at the threshold value of 1/12 vs. 1/6. The neutralizing antibody positivity against the Delta variant was higher in the TURKOVAC arm regardless of sex and threshold values, suggesting that TURKOVAC is more effective than CoronaVac against the Delta variant in both sexes. This can be explained, again, by the fact that TURKOVAC was produced in the period when the Delta variant had emerged and that heterologous booster doses might have produced higher immunogenicity, which requires further studies exploring the efficacy of homologous vs. heterologous booster doses.

The immune system is affected by age showing a decrease in immunity with aging, which is called immunosenescence [43,44]. Nevertheless, a meta-analysis reported no difference in vaccine efficacy between people of all ages [24]. Because of decreasing neutralizing antibody titers in older people [45], the WHO recommends a homologous or heterologous booster dose after the completion of primary vaccination in people aged ≥60 years [46]. In the present study, the neutralizing antibody positivity on Day 84 was comparable between the vaccine arms in all age groups, regardless of the variant. However, the neutralizing antibody positivity was slightly higher in the TURKOVAC arm in all but the 50–60 years age group, whereas the positivity was higher in the CoronaVac arm against both variants, suggesting that the homologous booster might be a better option for individuals in the 50–60 years age group, which needs to be confirmed through further studies. A modeling study from France found that a booster dose for people ≥65 years was the least effective in protecting people against hospitalization [27]. In the present study, the oldest age group consisted of people at the age of 50–60 years; therefore, we could not evaluate the immunogenicity of booster doses in the age group of ≥60 years.

A recent study from Türkiye compared the anti-spike IgG antibody response, to the booster dose of CoronaVac vs. BNT162b2, in healthcare professionals after primary vaccination with CoronaVac and found increased IgG spike levels in both vaccine arms [47]. This finding was supported in another study reporting higher antibody responses including anti-spike IgG antibodies after three doses vs. two doses of vaccine, which was not statistically significant. In this study, the authors found an association between age and anti-spike IgG antibody levels [48]. Contrarily, Uysal et al. [49] found no association between age and receptor-binding domain (RBD) of the SARS-CoV-2 spike protein. Bartsch et al. [50] demonstrated that anti-spike IgG antibodies induced by the vaccines are also effective against the Omicron variant.

We determined comparable anti-spike IgG antibody positivity between the vaccine arms in both sexes and in all age groups, with slightly lower responses in the 50–60 years age group as compared to other age groups, and in the TURKOVAC arm vs. the CoronaVac arm, suggesting the beneficial effect of homologous boosting in this age group, which again needs to be verified with further studies.

The percentage of participants with a ≥4-fold increase in the anti-spike IgG antibody titers 28 days after the booster dose was higher in the TURKOVAC arm than in the CoronaVac arm (82.5% vs. 64.9%), with a higher percentage of females than males showing a ≥4-fold increase in the IgG anti-spike antibody titer in both vaccine arms. 

Our results support the fact that a booster dose, either homologous or heterologous, induces immunity with increased neutralizing antibody and anti-spike IgG antibody positivity. Moreover, increased antibody positivity on Day 28 after the booster dose persists on Day 84. Although the vaccine arms were comparable, TURKOVAC showed more favorable effectiveness against both Wuhan and Delta variants than CoronaVac, regardless of sex and age. However, CoronaVac is more beneficial for people in the 50–60 years age group.

The relatively small sample size and relatively short follow-up period (84 days) are the major limitations of the present study. Furthermore, the exclusion of people over the age of 60 years, people who have comorbidities, as well as immunocompromised individuals, are the other limitations for drawing a definite conclusion about antibody positivity in certain groups. In addition, in the present study, we excluded patients who have been infected with SARS-CoV-2 and patients who became positive for COVID-19 after primary vaccination, in order to thoroughly evaluate the dynamics of vaccine response; this group of patients may also require a booster dose as neutralizing antibodies are likely to decrease over time. Moreover, the neutralizing antibody against the Omicron variant was not measured.

## 5. Conclusions

The TURKOVAC and CoronaVac vaccines are effective as booster doses in both sexes and in all age groups, with TURKOVAC producing slightly higher positivity against both Wuhan and Delta variants. However, boosting with CoronaVac seems to be more beneficial for those in the 50–60 years age group. Further randomized studies are needed to draw a conclusion regarding the target population, and the timing and number of booster doses, and to collect information about the necessity of integrating TURKOVAC into primary vaccination schedules in order to maintain immunity for a long time. In addition, studies comparing homologous and heterologous boosting with CoronaVac and TURKOVAC are required. 

## Figures and Tables

**Table 1:** Patient distribution according to age and sex quotas [10].

	Male		Female		
	18–39 Years	40–60 Years	18–39 Years	40–60 Years	Total
CoronaVac (n)	40	35	20	16	111
TURKOVAC (n)	40	35	20	16	111
Total	80	70	40	32	222

**Table 2:** Characteristics of the study participants.

		TURKOVAC	CoronaVac	Total	*p*
Sex	Female	34 (31.5)	38 (33.3)	72 (32.4)	0.768
	Male	74 (68.5)	76 (66.7)	150 (67.6)	
	Total	108 (100)	114 (100)	222 (100)	
Age groups, years	18–29	13 (12.0)	20 (17.5)	33 (14.9)	0.587
	30–39	44 (40.7)	39 (34.2)	83 (37.4)	
	40–49	37 (34.3)	38 (33.3)	75 (33.8)	
	50–60	14 (13.0)	17 (14.9)	31 (14.0)	
	Total	108 (100)	114 (100)	222 (100)	
COVID-19 (+)	No	84 (77.8)	83 (72.8)	167 (75.2)	0.391
	Yes	24 (22.2)	31 (27.2)	55 (24.8)	
	Total	108 (100)	114 (100)	222 (100)	
COVID-19 (+) participants after the booster dose	Day 0–28	4	3	4	
	Day 28–84	7	7	14	
	Day ≥ 84	13	24	37	

**Table 3:** Distribution of participants with a 4-fold increase in anti-spike IgG antibody titers according to sex.

		TURKOVAC	CoronaVac	Total	*p*
		n (%)	n (%)	n (%)	
Female	<4-fold	3 (9.1)	11 (28.9)	14 (19.7)	0.036
	≥4-fold	30 (90.9)	27 (71.1)	57 (80.3)	
Male	<4-fold	15 (21.4)	29 (38.2)	44 (30.1)	0.028
	≥4-fold	55 (78.6)	47 (61.8)	102 (69.9)	
Total	103 (47.46)	114 (52.53)	217 (100)		

## Data Availability

The data presented in this study are available on request from the corresponding author.

## Supplementary Materials

The following supporting information can be downloaded at: https://www.mdpi.com/2076-393X/11/7/1234/s1, Supplementary Material S1: Neutralizing antibody positivity against the Wuhan variant according to age and sex groups at the threshold value of ≥1/12; Figure S1: Neutralizing antibody positivity against the Wuhan variant in the TURKOVAC and CoronaVac arms at the threshold value of 1/12; Figure S2: Neutralizing antibody positivity against the Wuhan variant in the TURKOVAC and the CoronaVac vaccine arms at the threshold value of ≥1/12 (**a**) in females; (**b**) in males; Figure S3: Neutralizing antibody positivity against the Wuhan variant in the TURKOVAC and CoronaVac arms at the threshold value of ≥1/12 in the (**a**) 18–29 years age group; (**b**) 30–39 years age group; (**c**) 40–49 years age group; (**d**) 50–60 years age group; Supplementary Material S2: Neutralizing antibody positivity against the Delta variant according to age and sex groups at the at the threshold value of ≥1/12; Figure S4: Neutralizing antibody positivity against the Delta variant in TURKOVAC and CoronaVac arms at the threshold value of ≥1/12; Figure S5: Neutralizing antibody positivity against the Delta variant in the TURKOVAC and CoronaVac vaccine arms at the threshold value of ≥1/12 (**a**) in females; (**b**) in males; Figure S6: Neutralizing antibody positivity against the Delta variant in the TURKOVAC and the CoronaVac vaccine groups at the threshold value of ≥1/12 in the (**a**) 18–29 years age group; (**b**) 30–39 years age group; (**c**) 40–49 years age group; (**d**) 50–60 years age group.
