# Supplementary material for: Long-Term Results of Immunogenicity of Booster Vaccination against SARS-CoV-2 (Hybrid COV-RAPEL TR Study) in Turkiye: A Double-Blind, Randomized, Controlled, Multicenter Phase 2 Clinical Study
Source: Vaccines (Basel). 2023 Jul 12;11(7):1234. doi: 10.3390/vaccines11071234 (PMC10416156; doi:10.3390/vaccines11071234)
Supplement: Supplementary file 1 [file vaccines-11-01234-s001.zip › Supplemantary Material S2-proofread-11.07.2023.pdf]

SUPPLEMENTARY MATERIAL S2: Neutralizing antibody positivity against the Delta variant according to age and sex groups at the threshold value of ≥1/12.

The neutralizing antibody positivity against the Delta variant at the threshold value of ≥1/12 decreased on Day 84 vs. Day 28 in the TURKOVAC arm and slightly increased on Day 84 vs. Day 28 in the CoronaVac arm (Figure S4).

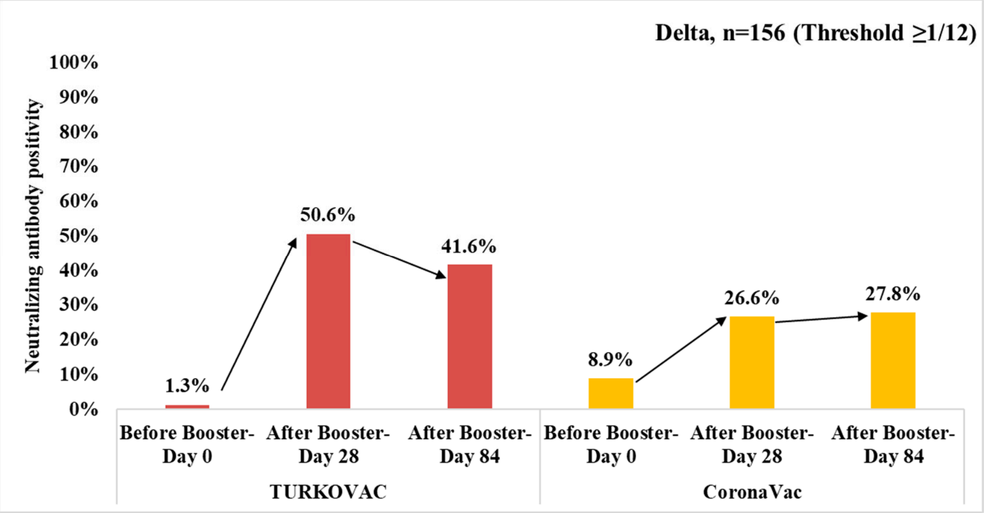

Figure S4. Neutralizing antibody positivity against the Delta variant in TURKOVAC and CoronaVac arms at the threshold value of ≥1/12.

In females, the neutralizing antibody positivity against the Delta variant at the threshold value of ≥1/12 decreased on Day 84 vs. Day 28 in the TURKOVAC arm, whereas it increased on Day 84 vs. Day 28 in the CoronaVac arm (Figure S5a). In males, the antibody positivity slightly decreased on Day 84 vs. Day 28 in both vaccine arms (Figure S5b). Although the neutralizing antibody positivity showed an increasing trend on Day 84 in the CoronaVac arm in females, it was lower than the TURKOVAC arm in both sexes.

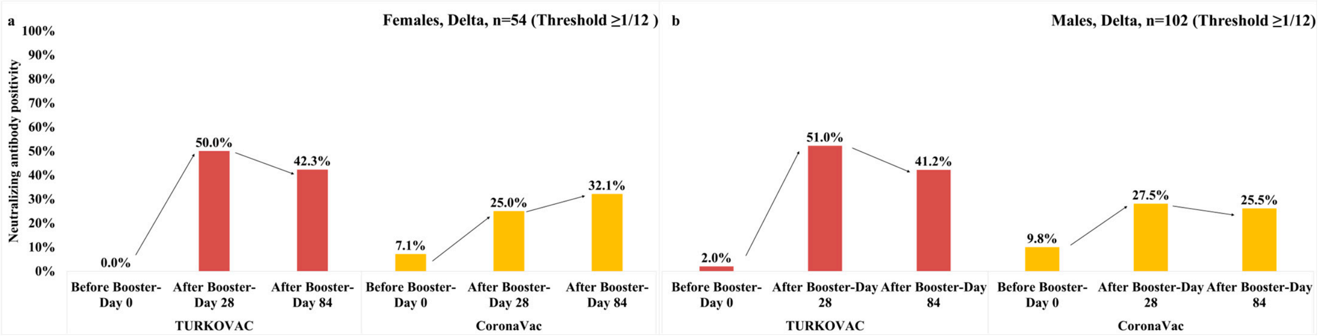

Figure S5. Neutralizing antibody positivity against the Delta variant in the TURKOVAC and CoronaVac vaccine arms at the threshold value of ≥1/12 **(a)** in females; **(b)** in males.

At the threshold value of ≥1/12, the neutralizing antibody positivity against the Delta variant increased on Day 28 and remained the same on Day 84 in the TURKOVAC arm, whereas it continued to increase until Day 84 in the CoronaVac arm in the 18-29 years and 30-39 years age groups. In both age groups, the neutralizing antibody positivity on Day 84 was higher in the TURKOVAC arm (Figures S6a and S6b). Both in the 40-49 years and 50-60 years age groups, the antibody positivity slightly decreased on Day 84 in both vaccine arms; however, antibody positivity on Day 84 was higher in the TURKOVAC arm

(Figures S6c and S6d). The neutralizing antibody positivity on Day 84 was higher in all age groups in the TURKOVAC arm vs. the CoronaVac arm at the threshold value of ≥1/12.

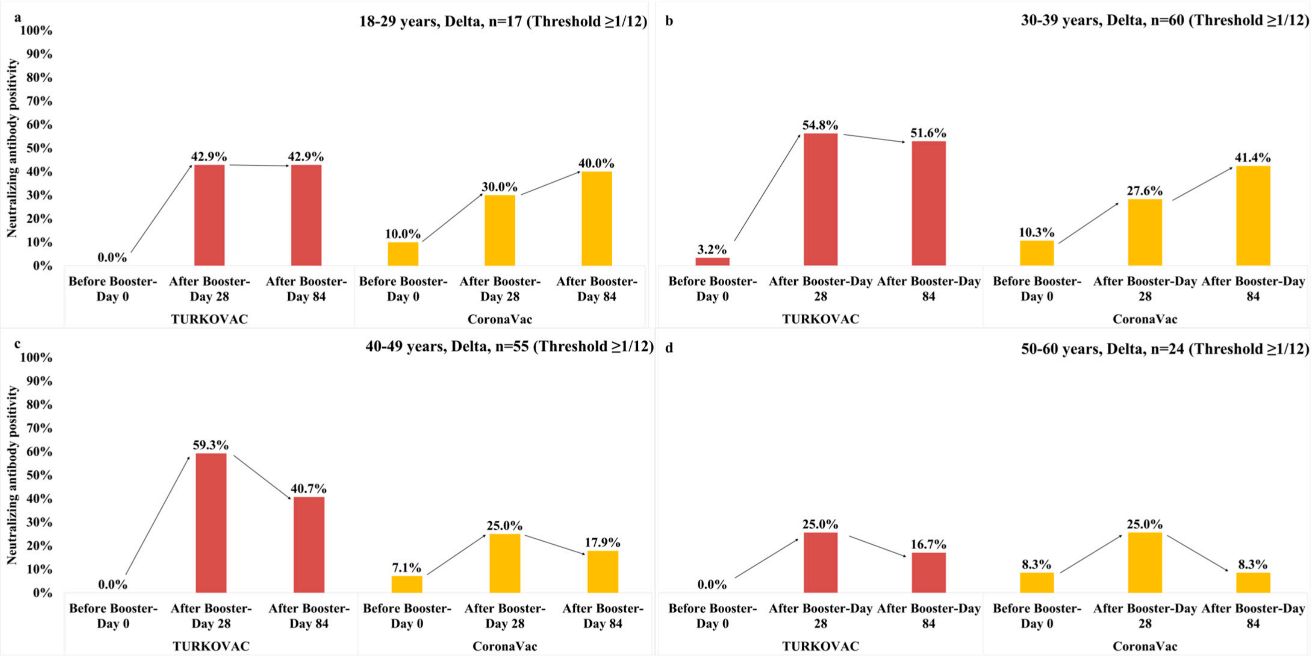

Figure S6. Neutralizing antibody positivity against the Delta variant in the TURKOVAC and the CoronaVac vaccine groups at the threshold value of ≥1/12 in the **(a)** 18-29 years age group; **(b)** 30-39 years age group; **(c)** 40-49 years age group; **(d)** 50-60 years age group.
