# Supplementary material for: Long-Term Results of Immunogenicity of Booster Vaccination against SARS-CoV-2 (Hybrid COV-RAPEL TR Study) in Turkiye: A Double-Blind, Randomized, Controlled, Multicenter Phase 2 Clinical Study
Source: Vaccines (Basel). 2023 Jul 12;11(7):1234. doi: 10.3390/vaccines11071234 (PMC10416156; doi:10.3390/vaccines11071234)
Supplement: Supplementary file 1 [file vaccines-11-01234-s001.zip › Supplementary Material S1-proofread-11.07.2023.pdf]

SUPPLEMENTARY MATERSIAL S1: Neutralizing antibody positivity against the Wuhan variant according to age and sex groups at the at the threshold value of ≥1/12.

The neutralizing antibody positivity against the Wuhan variant at the threshold value of ≥1/12 increased on Day 84 vs. Day 28 in the TURKOVAC arm, whereas it slightly decreased on Day 84 vs. Day 28 in the CoronaVac arm (Figure S1).

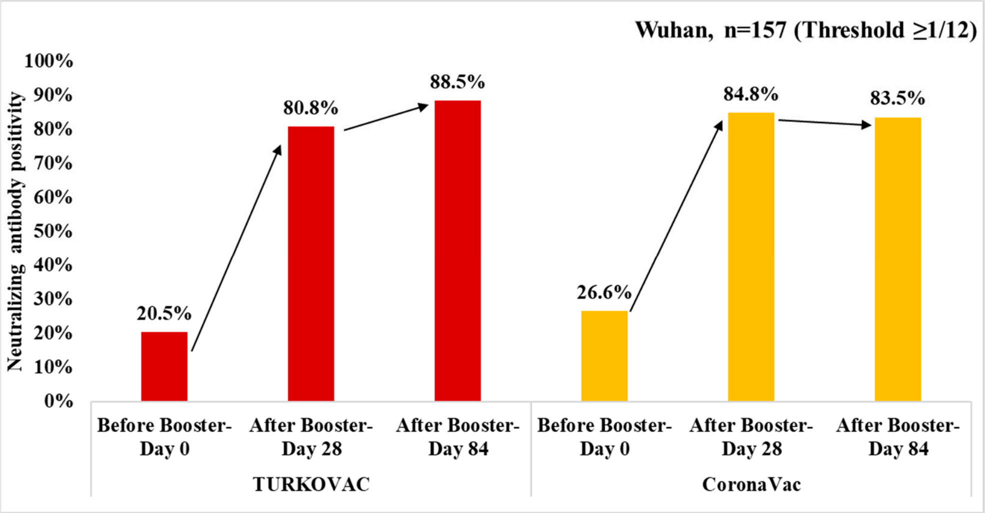

Figure S1. Neutralizing antibody positivity against the Wuhan variant in the TURKOVAC and CoronaVac arms at the threshold value of 1/12.

The neutralizing antibody positivity against the Wuhan variant at the threshold value of ≥1/12 slightly increased on Day 84 vs. Day 28 in females in the TURKOVAC arm, whereas it was slightly decreased on Day 84 vs. Day 28 in the CoronaVac arm (Figure S2a). The neutralizing antibody positivity against the Wuhan variant in males slightly increased on Day 84 vs. Day 28 in both vaccine arms (Figure S2b).

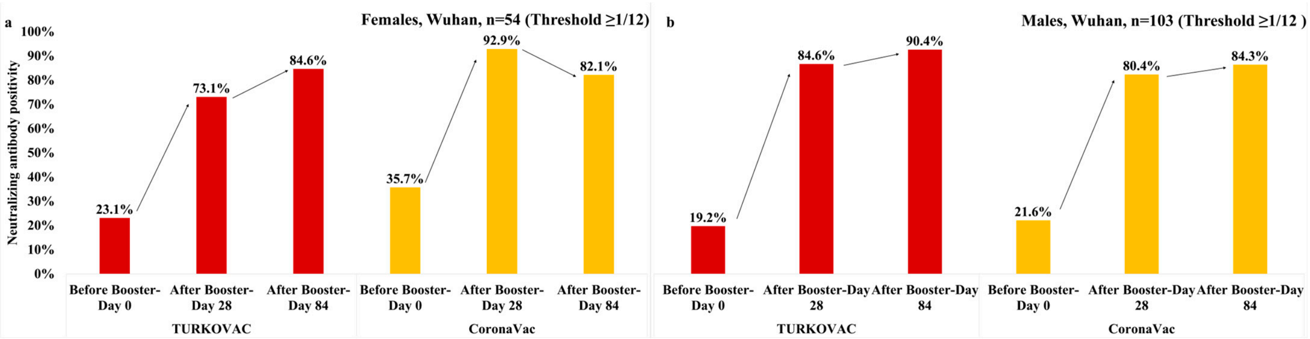

Figure S2. Neutralizing antibody positivity against the Wuhan variant in the TURKOVAC and the CoronaVac vaccine arms at the threshold value of ≥1/12 (**a**) in females; (**b**) in males.

At the threshold value of ≥1/12, the neutralizing antibody positivity against the Wuhan variant in the 18-29 years age group increased on Day 28 and remained same on Day 84 in the TURKOVAC arm, whereas it increased on Day 28 and then slightly decreased on Day 84 in the CoronaVac arm (Figure S3a). In the 30-39 years age group, the neutralizing antibody positivity increased on Day 28 and continued to increase until Day 84 in both vaccine arms; however, the increase on Day 84 vs. Day 28 was slightly higher in the TURKOVAC arm (Figure S3b). In the 40-49 years age group, the neutralizing

antibody positivity increased on Day 28 and continued increasing until Day 84 in the TURKOVAC arm, whereas it slightly decreased on Day 84 vs. Day 28 in the CoronaVac arm (Figure S3c). In the 50-60 years age group, the antibody positivity increased on Day 28 in both vaccine arms, and it continued to increase and reached to the maximum level on Day 84 in the CoronaVac arm, whereas it decreased on Day 84 in the TURKOVAC arm (Figure S3d).

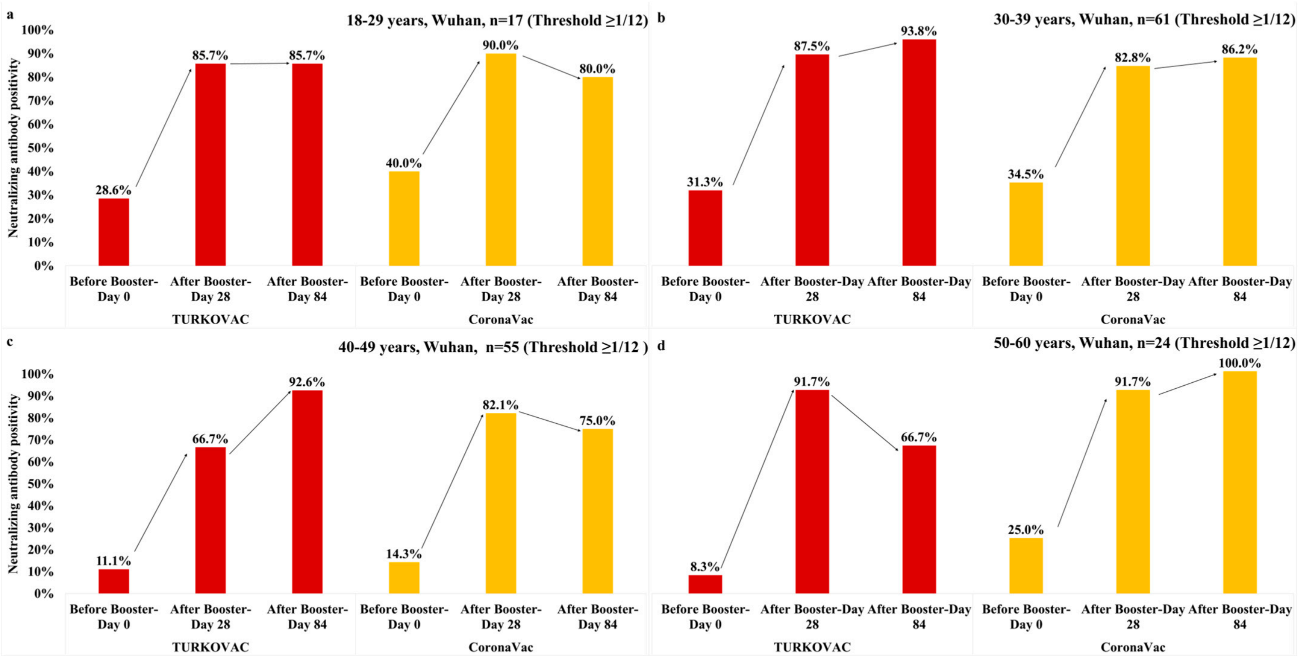

Figure S3. Neutralizing antibody positivity against the Wuhan variant at the threshold value of ≥1/12 in the TURKOVAC and CoronaVac arms in the **(a)** 18-29 years age group; **(b)** 30-39 years age group; **(c)** 40-49 years age group; **(d)** 50-60 years age group.
